# The relationship between irritability, depression and anxiety among Chinese college students during the COVID-19 pandemic: A network analysis

**DOI:** 10.3389/frcha.2023.1045161

**Published:** 2023-04-06

**Authors:** Ling Li, Lei Ren, Xiaoqing Zhan, Lingzhi Wang, Chang Liu, Mengxue Zhao, Xi Luo, Zhengzhi Feng, Kuiliang Li

**Affiliations:** ^1^ Department of General Education, Chongqing Water Resources and Electric Engineering College, Chongqing, China; ^2^ Department of Psychology, Fourth Military Medical University, Xi'an, China; ^3^ Medical English Department, College of Basic Medicine, Army Medical University, Chongqing, China; ^4^ Sichuan Shun Dao Law Firm, Chengdu, China; ^5^ BrainPark, Turner Institute for Brain and Mental Health and School of Psychological Sciences, Monash University, Clayton, VIC, Australia; ^6^ School of Psychology, Army Medical University, Chongqing, China

**Keywords:** depression, anxiety, comorbidity, network analysis, irritability

## Abstract

**Introduction:**

Irritability, a common symptom included in the 5th Edition of Diagnostic and Statistical Manual of Mental Disorders (DSM-5), is thought to be associated with multiple emotional disorders. It is commonly seen among college students in isolation during the COVID-19 pandemic. However, its relation with anxiety and depression remains unclear. We aim to study the relation of irritability, anxiety and depression in Chinese college students during the COVID-19 pandemic by using network analysis to understand the co-occurrence of these three disorders.

**Methods:**

During the COVID-19 pandemic, we recruited 1516 college students from five general universities in China to complete the Irritability, Depression and Anxiety Scale (IDA-S) to analyze the symptom network of irritability, depression and anxiety. Specifically, we assessed the indices of strength centrality and bridge strength for each node in the network.

**Results:**

Some strongest linkages were found among anxiety symptoms “nervous” and “panic” (weight = 0.36), depression symptoms “sad mood” and “amused” (weight = 0.32), inward irritability items “self-hurt” and “self-harm” (weight = 0.32) and outward items “rough” and “aggressive” (weight = 0.28). The anxiety symptom “panic” had the highest strength value, followed by the inward irritability symptom “annoyed”. The nodes “ease” and “sleep” had the lowest strength value. In addition, the anxiety symptom “relax” had the highest bridge strength value, followed by inward irritability symptom “annoyed”.

**Conclusion:**

This study explored the characteristics of a network of irritability, depression and anxiety symptoms among Chinese college students during the COVID-19 pandemic. We found that anxiety and irritability symptoms played an important role in the network. The findings provide evidence for prevention and intervention for college students' mental health problems during the COVID-19 pandemic.

## 1. Introduction

The number of SARS-Cov-2 infections continues to rise, as does the number of deaths, resulting in a significant psychological impact on the public. In order to prevent the spread of COVID-19 pandemic, people are quarantined or asked to stay at home or to keep a social distance in China, which increases the risk of mental disorders (1) such as anxiety, depression (2, 3) and fearmongering (4). Therefore, in the wake of the COVID-19 outbreak, there is an urgent call for psychological crisis intervention. Previous studies on the psychological responses suggest that psychological crisis intervention plays a key role in epidemics and crises, such as the Wenchuan earthquake, SARS epidemic outbreak in China (5, 6), or the September 11 attacks in the United States (7). As early as the end of January 2020, the National Health Commission of China issued a notice on the guiding principles of emergency psychological crisis intervention for the outbreak of the COVID-19 pandemic. Nevertheless, we are facing difficulties in implementing psychological interventions in China, such as an inadequate integrated plan and a lack of professional and technical personnel (8). Therefore, up to now few people have been intervened psychologically, including the college students.

The negative emotions caused by the COVID-19 pandemic include irritation, anxiety, and depression (9), but it is unclear what relationship exists among irritability, depression and anxiety in Chinese college students during the COVID-19 pandemic. The spread of the COVID-19 pandemic brought fear, anxiety, and worry, making people experience feelings of dread and despair in the world (10). In addition, social isolation has caused more psychological problems (4). Investigations confirmed that many college students during the COVID-19 pandemic suffer from anxiety (11), depression (12), and irritability (13), especially depression. Furthermore, the current available evidence showed an increase in depression and anxiety among college students during the COVID-19 pandemic (14, 15). In China, there are only 1.53 psychiatrists per 100,000 people, and the population with depression accounts for 3.02%, which does not include the number of missed diagnoses (16), indicating that a large number of people cannot get psychological counseling. Therefore, exploring the complex relationship between irritability, anxiety and depressive symptoms in college students is beneficial for the prevention of these disorders.

Anxiety and depression are often accompanied by varying degrees of irritability, which is common among the symptoms of DSM-5. Although irritability is not regarded as an independent disorder category in DSM-5, it is closely related to many disorders, such as generalized anxiety disorder, depression disorder, post-traumatic stress disorder and schizoaffective disorder (17, 18). Irritability is also a common symptom in severe mood dysregulation. Previous research suggests that irritability is a specific predictor of anxiety and depression disorder in youth (19). Moreover, longitudinal data indicate that adolescent non-onset irritability is common and is associated with an increased risk of adult anxiety and unipolar depression disorder, rather than bipolar disorder (20). Studies have shown that increasing the severity of irritability can increase the lifetime prevalence of depression by 0.4%, and persistent irritability will increase the prevalence by an additional 0.2% (21). Youths with anxiety disorder report more severe irritability than healthy people, so do their parents (22). Genetic evidence indicates that irritability is a crucial factor for predicting anxiety and depression, and heritable genetic factors and unique environmental factors are important factors for irritability, anxiety and depression symptoms (23), indicating that irritability is closely related to anxiety and depression disorders.

In summary, irritability, depression and anxiety are the main factors of common neuropsychiatric disorders, and play an important role in clarifying the relation between disorders (24). The relation between depression and anxiety has long been considered controversial. The overlapping of symptoms associated with these disorders makes diagnosis, research, and treatment particularly difficult (25). Studies have shown that depression and anxiety coexist in up to 25% of all general patients. About 85% of patients with depression suffer from severe anxiety, and 90% of patients with anxiety suffer from depression (26). Therefore, researchers began to focus on the comorbidity of anxiety and depression (27) and their treatment (28). However, irritability has been rarely studied as the main symptom to explore the relation between anxiety and depression.

Network analysis is an important approach to exploring the symptom-to-symptom relation, and the network model is useful to assess the importance of each symptom in a disorder (29). The network is composed of nodes and edges, with nodes representing variables and edges representing the correlation between two variables. Centrality is often used to assess the importance of the network, including strength, closeness and betweenness (30). Recent studies have shown that strength centrality is more stable than closeness and betweenness centrality which seems especially unsuitable as assessments of node importance in psychological networks (31, 32). In addition, researchers proposed a concept of the initial bridge symptoms, which are considered to be overlapping symptoms of two mental illnesses (33). Jones et al. (34) further studied the bridge centrality indices, confirming that the elimination of bridge symptoms in comorbidity networks is more effective than the elimination of traditional central nodes. In addition, compared with empirical network research, the network model based on strength estimation can explain the symptoms with a higher strength because of more reliable indicators of latent variables (35). In view of this, we assessed the centrality of the strength and the bridge strength in the present study.

A previous study examining the network structure of anxiety and depressive symptom among college students during the COVID-19 pandemic confirmed that irritability, uncontrollable worry, trouble relaxing, and depressed mood had the highest centrality values (36). Depressed affect emerged as a robust central symptom and bridge symptom across Anxiety-Depression networks (37). At present, however, there are few studies to explore comorbidity networks related to irritability. Because irritability is closely related to anxiety and depression, it is pretty important to explore the comorbidity network among them, which will provide a new insight into understanding the comorbidity among the three disorders. In the present study, we computed the comorbidity network to investigate the relations among internal symptoms: irritability, depression and anxiety. In addition, we assessed the importance of each symptom in the comorbidity network by using strength centrality and the extent for promoting activations to spread from one cluster to others by using bridge strength centrality. The following hypotheses were proposed:

Hypothesis 1: There is a strong relationship between irritability, depression and anxiety symptoms in college students during COVID-19 pandemic.
Hypothesis 2: Depression symptoms are the most important symptom in college students during COVID-19 pandemic.
Hypothesis 3: The irritability symptoms play an important role in the irritability, depression and anxiety network.

## 2. Methods

### 2.1. Ethics statement

This research received an ethical review from the Department of Medical Psychology, Army Medical University (Project No. CWS20J007). Participants were aware of the informed consent before participation in this study, were informed that the survey was anonymous, and were assured that personal information would not be disclosed.

### 2.2. Participants

This study was part of the “Mental Health during COVID-19” project, and the investigations were conducted online by Questionnaire Star due to the COVID-19 lockdown. The participants were recruited by posting questionnaire on social software such as QQ and WeChat. The initial instruction provided a brief description of the investigation and informed that the investigation is only for research purposes, and all data will be kept confidential and anonymous. Participants were required to answer all the questions. We also randomly distributed 1 to 5 RMB remuneration after the questionnaire survey.

The data came from an online survey including students of all grades from five general universities in China. A total of 1,554 students participated in the questionnaire survey. Based on the length of the questionnaire (includes 48 items), the average time for answering each question is 4 s. Therefore, we set a threshold for the minimum time of completing the questionnaire, that is 200 seconds. To ensure the accuracy of the data, we deleted the data of 38 participants who took less than 200 s to complete the questionnaire or who were under the age of 18 (because they were considered minors in China), and finally included 1,516 participants aged from 18 to 23 years (M = 20.2, SD = 1.66) (Table 1). The efficiency rate of the survey was 97.62%.

**Table 1:** Sample characteristics (*N* = 1516).

	*n* (%) or mean (S.D.)
Sociodemographics
Mean age, years (S.D.)	20.2 (1.66)
Men, *n* (%)	352 (23.2)
Emotion disorders, *n* (%)
Depression, > 3	621 (41.0)
Anxiety, > 5	783 (51.6)
Inward irritability, > 3	601 (39.6)
Outward irritability, > 4	426 (28.1)

S.D., standard deviation; Based on descriptive statistics.

The score is based on the cut-off criteria for IDAs.

### 2.3. Measures

The Irritability, Depression and Anxiety Scale was adopted in the survey (38). It is a 4-point (ranging from 0 to 3) Likert scale, containing 4 dimensions: depression, anxiety, inward irritability and outward irritability, and involving 18 items. Previous studies showed that the questionnaire has good a credibility (39, 40). The Cronbach's *α* of the Chinese version was 0.85 in the current study (Tables 2, 3).

**Table 2:** Item abbreviations.

Abbreviation	IDA-S item(s)
Sad mood	I feel cheerful. (DEPRESSION)
Relax	I can sit down and relax quite easily. (ANXIETY)
Appetite*	My appetite is good? (DEPRESSION)
Rough*	I lose my temper and shout or snap at others. (OUTWARD IRRITABILITY)
Nervous*	I feel tense or “wound up”. (ANXIETY)
Self-harm*	I feel like harming myself. (INWARD IRRITABILITY)
Anhedonia	I have kept up my old interests. (DEPRESSION)
Patient	I am patient with other people. (OUTWARD IRRITABILITY)
Panic*	I get scared or panicky for no very good reason. (ANXIETY)
Angry*	I get angry with myself or call myself names. (INWARD IRRITABILITY)
Amused	I can laugh and feel amused. (DEPRESSION)
Aggressive*	I feel I might lose control and hit or hurt someone. (OUTWARD IRRITABILITY)
Disgust*	I have an uncomfortable feeling like butterflies in the stomach. (ANXIETY)
Self-hurt*	The thought of hurting myself occurs to me. (INWARD IRRITABILITY)
Sleep*	I’m awake before I need to get up. (DEPRESSION)
Upset*	People upset me so that I feel like slamming doors or banging about. (OUTWARD IRRITABILITY)
Ease	I can go out on my own without feeling anxious. (ANXIETY)
Annoyed*	Lately I have been getting annoyed with myself. (INWARD IRRITABILITY)

IDA-S, irritability, anxiety and depression scale, including 4 subscales of depression, anxiety, inward irritability and outward irritability.

* Represents reverse item.

**Table 3:** Descriptive statistics of each item (*n* = 1516).

Item	M	SD	Pre
1. Sad mood	0.82	0.69	38%
2. Appetite	1.00	0.62	20%
3. Amused	0.51	0.63	42%
4. Sleep	0.32	0.74	9%
5. Anhedonia	0.64	0.66	27%
6. Relax	0.76	0.71	34%
7. Disgust	1.25	0.87	22%
8. Nervous	1.56	0.72	34%
9. Panic	1.18	0.83	44%
10. Ease	0.86	0.94	3%
11. Self-hurt	0.79	0.93	43%
12. Self-harm	0.82	0.85	45%
13. Angry	0.58	0.81	34%
14. Annoyed	0.89	0.75	43%
15. Rough	0.79	0.81	38%
16. Aggressive	0.55	0.79	40%
17. Patient	1.30	0.60	13%
18. Upset	0.70	0.77	33%

M, mean; SD, standard deviation; Pre, predictability.

.

### 2.4. Data analysis

We sorted out raw data using Excel software, and used R software (version 4.0.3) and its packages (including *qgraph*, *networktools*, *mgm*, *bootnet*) for data analysis and graphical visualization. We first estimated the network structure of irritability, depression and anxiety (IDA) of the college students, then estimated node strength centrality, bridge strength centrality and predictability indices, and after that assessed the stability of these indices. The detailed steps of the analysis are described below.

Gaussian Graphical Model (GGM) was used to estimate the networks by the R package *qgraph* (41, 42). GGMs are undirected networks and the edges represent partial correlations between two connected nodes after statistically controlling for all other nodes in the network (43). The estimations of GGM were based on nonparametric Spearman rho correlation matrices. Table S1 (Supplementary Materials Table S1) showed the nonparametric Spearman rho correlation matrix of the selected nodes for the present network. We used graphical least absolute shrinkage and selection operator (LASSO) algorithm to regularize the GGM. This step shrinks all edges and causes small edges to become zero to obtain a more stable and interpretable network (44). The GGM tuning parameter was set to the suggested value of 0.5 to well judge and weigh the sensitivity and specificity of finding out true edges (45). In the visualized networks, red edges represented negative partial correlations, and blue edges represented positive partial correlations between nodes. Thicker edges represented stronger correlations between nodes.

The importance of each node in the present network was assessed *via* the strength centrality in the R package *qgraph* (42). The strength centrality of a given node is defined as the sum of the absolute value of all edges attaching to this node. Higher-strength centrality values indicate a greater importance in the network. Then, we estimated the bridge strength centrality using the bridge function *via* the R package *networktools* (34). Bridge strength centrality is defined as the sum of the absolute value of all edges that connect a given node and the nodes in the other clusters (34). The nodes in the present network were separated *a priori* into four clusters: a cluster of depression symptoms (i.e., sad mood, appetite, amused, sleep, and anhedonia), a cluster of anxiety symptoms (i.e., relax, disgust, nervous, panic, and ease), a cluster of inward irritability symptoms (i.e., self-hurt, self-harm, angry, and annoyed), and a cluster of outward irritability symptoms (i.e., rough, aggressive, patient, and upset). Higher bridge strength centrality values indicate a greater extent for promoting activations to spread from one cluster to others. In addition, the predictability of each node was calculated *via* the R package *mgm* (46). Predictability is defined as the variance in a node that is explained by all its neighboring nodes.

The robustness of the network was examined *via* the R package *bootnet* (47). First, the accuracy of edge weights was evaluated by constructing 95% confidence intervals (CIs) using a non-parametric bootstrap procedure (2,000 bootstrap samples). Second, the stability of node strengths and bridge strengths was evaluated by calculating the correlation stability (CS) coefficient using a case-dropping bootstrap procedure. The value of CS-coefficient should not be below 0.25 and preferably should be above 0.5 (47). Third, bootstrapped difference tests (2,000 bootstrap samples and *α* = 0.05) for edge weights, node strengths and node bridge strengths were performed to confirm whether two edge weights, two-node strengths, or two-node bridge strengths differ significantly from one another. The results of the robustness analyses for the present network are shown in the Supplementary Materials Figures S1-S6).

## 3. Results

### 3.1. Descriptive statistics

A total of 1,554 questionnaires were collected in this study, among which 1,516 were retained for data analysis, with a response rate of 97.62%. Among the participants, 352 were boys, accounting for 23.2% of the total. The participants aged from 18 to 23 (M = 20.2, SD = 1.66) (Table 1).

### 3.2. Network structure

**Figure 1:**
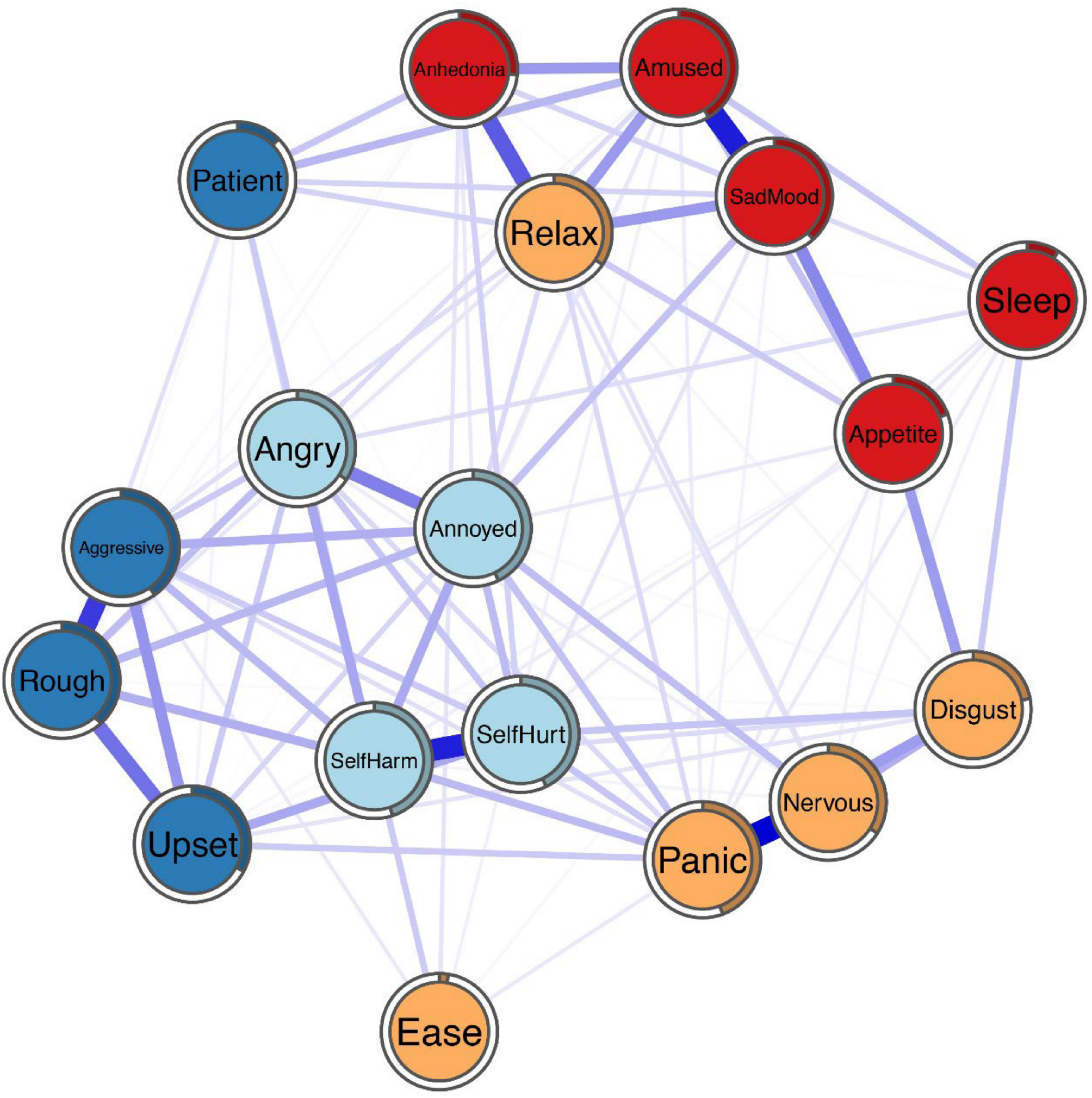
Network structure of irritability, depression and anxiety symptoms in the participants during the epidemic isolation.

The comorbidity network is depicted in Figure 1. There are several obvious characteristics. First, except for items “relax”, “ease” and “patient”, each item was distributed according to the dimension to which it belongs. Second, 101 (66.0%) were non-zero out of 153 possible edges, which were all positive. Third, some strong linkages were found among “nervous” and “panic” (weight = 0.36), “sad mood” and “amused” (weight = 0.32), “self-hurt” and “self-harm” (weight = 0.32), “rough” and “aggressive” (weight = 0.28), “relax” and “anhedonia” (weight = 0.23), and “rough” and “upset” (weight = 0.20). Fourth, the rates of node predictability ranged from 3% to 45% with an average of 31% (Table 3). The item “ease” had the lowest predictability sharing 3% of the variance with its neighboring nodes. The item “self-harm” had the highest predictability sharing 45% of the variance with its neighboring nodes. The items of node predictability rate more than 40% were “panic” (44%), “self-hurt” (43%), “annoyed” (43%), and “amused” (42%).

**Figure 2:**
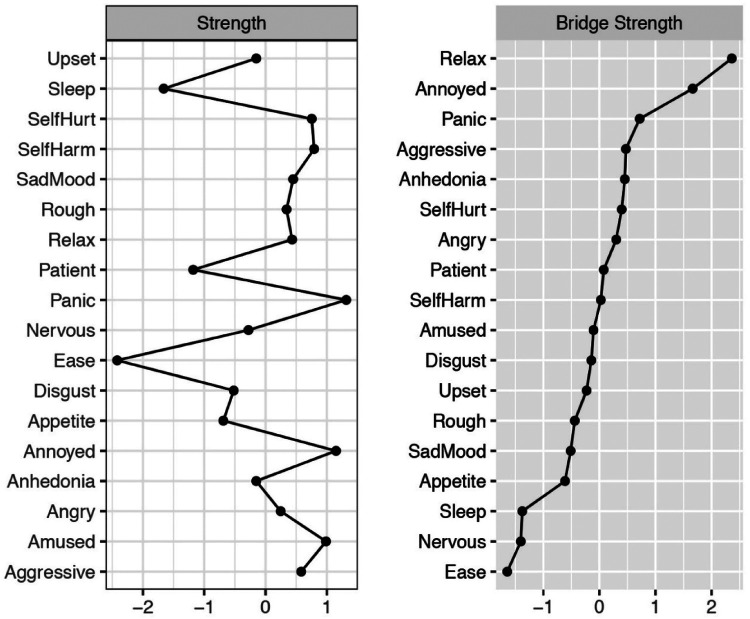
Node strength centrality and bridge strength centrality in network analysis of irritability, depression, anxiety symptoms.

The value (*z*-scored) of strength and bridge strength for each node is depicted in Figure 2. The item “panic” had the highest strength value, indicating that it is the most connected node in the network, which is followed by “annoyed”. Two nodes with the lowest strength value were “ease” and “sleep”. In addition, the item “relax” had the highest bridge strength value, which was followed by “annoyed”. Three nodes with the lowest bridge strength value were “ease”, “nervous” and “sleep”. Bootstrapped 95% CIs indicating the accuracy of edge weights was confirmed (see Supplementary Materials Figure S1). In terms of the stability of centrality indices, CS-coefficients of node strength and bridge strength were both 0.75 which can be considered as highly stable (see Supplementary Materials Figure S3 and Supplementary Materials Figure S5). Bootstrapped difference test for edge weights showed a moderate proportion of differences among edge weights that were significant in the present network (see Supplementary Materials Figure S2). Supplementary Materials Figure S4 and Supplementary Materials Figure S6 depict bootstrapped difference test for node strengths and bridge strengths (in Supplementary Materials). These results reveal that a large proportion of the differences among both node strengths and bridge strengths are significant in the present network.

## 4. Discussion

This study explored the linkage between irritability, depression and anxiety symptoms among college students in the network model. The network shows a strong interconnection and clusters were found within each symptom. Consistent with previous studies, the symptoms in the network are directly or indirectly connected (48, 49). However, the symptoms of anxiety and depression in this study do not share a one-to-one match with those in previous studies, indicating that symptoms of anxiety and depression have a more comprehensive link. It is impossible to cover all symptoms in a few polls, but more comprehensive symptoms are supposed to be considered in the comorbidity network of anxiety and depression, because they have connections in networks to a certain extent. Therefore, if irritability symptoms are excluded in pathophysiology studies of anxiety and depression, some significant findings may not be found.

The study found that the symptoms, “nervous” and “panic”, “sad mood” and “amused”, have significantly higher edge strength than others in the network. Consistent with previous studies, the strongest edge link in the network is among the edges of the symptoms within one disorder, but not between the disorders (48, 50). Beard et al. (2016) (48) found that the linkage between “relax” and other anxiety symptoms (i.e., restless) was stronger than that with depression symptoms in the network (48). Interestingly, inconsistent with Beard's study, the depression items “anhedonia” and “sad mood” and the anxiety item “relax” have the strongest linkages between the two disorders. It was found in the current study that the linkage between the anxiety item “relax” and depression items “sad mood” and “anhedonia” was stronger than that with other anxiety items. In addition, the edge link between the depression item “amused” and the outward irritability item “patient” is the strongest between the two disorders; meanwhile, the inward irritability item “self-harm” and the anxiety item “panic” have the strongest edge strength between the two disorders, which indicates that these nodes have the greatest connection between disorders.

In this network, the anxiety symptom “panic” had the highest strength, making it a hallmark symptom for anxiety among college students during the pandemic, inconsistent with the results of previous studies on anxiety and depression comorbidity network (48, 50). Their research found that “fatigue” and “sad mood”, which had the highest strength in the anxiety and depression comorbidity network, are the hallmark symptoms for depressive disorders. During the COVID-19 pandemic, the symptom “panic” had the highest strength which can be attributed to two possible reasons: first, college students' life and study were seriously affected; second, they worried too much about the risk of SARS-Cov-2 infection for themselves or their family members, which increases their anxiety (11, 51). Therefore, during the pandemic they have more anxiety symptoms “panic” and “worry” than depression symptoms. In addition, it is found that the inward irritability symptom “annoyed” had a higher strength in the network, inconsistent with the previous study which ignored the item “annoyed” (50). The symptom “annoyed” has higher strength in the network, indicating that it is more closely connected with other symptoms, which is likely due to the isolation during the pandemic. During isolation, the individuals' freedom of action is restricted and their cognitive ability is reduced (52, 53), which may reduce individuals' self-worth, resulting in the symptom “annoyed” or causing depression or anxiety. These results are inconsistent with our hypothesis 2 that depressive symptoms are not the most important symptom in the network; however, strong links remain between the nodes.

Inconsistent with previous studies where the main bridge symptoms are depression items “sad mood” and “anhedonia” (34, 50), there are two bridge symptoms in this network, namely “relax” and “annoyed”, which are considered to be more strongly connected to other depression symptoms than irritability and anxiety symptoms. This result is generally consistent with our hypothesis 3. One possible explanation is that current data are collected in forced isolation; therefore, these college students experienced greater anxiety than depression in isolation. Another explanation is that previous studies explored the comorbidity network of depression and anxiety, instead of irritability, depression and anxiety. Our research suggests that following an increase in irritability, “relax” becomes a more crucial factor in the network. Another higher strength bridge symptom is inward irritability item “annoyed”, inconsistent with the previous study (48), where irritability symptoms are not an important node in the network. Moreover, Garabiles' research showed that the anxiety symptom “irritability” is more strongly connected to depression items “anhedonia” and “fatigue” than other anxiety symptoms in the network (50), although in their research irritability is only considered as one of the anxiety symptoms. In summary the irritability item “annoyed” needs to be considered among college students during the pandemic (1) due to reduced chances to take part in social activities and less social support.

The innovation of this study lies in exploring the comorbidity network among irritability, depression and anxiety during the COVID-19 pandemic. These results may facilitate an effective psychological intervention, preventing individuals from developing mental illness which may increase the risk of contracting pneumonia (54). Another report also discussed the negative impact of mental illness on the spread of COVID-19 (55). However, due to the isolation in the pandemic, early diagnosis and prevention of depression and anxiety are difficult. The current psychological counseling is mainly initiated by individuals who actively seek help from the online system (56), while a large portion of potential patients may not receive the counseling. Moreover, because of a lack of psychiatrists, it is impossible to provide psychological counseling to every isolated person. To solve these problems, three psychological self-help suggestions are proposed as follows accordingly: 1) For the symptoms “nervous” and “panic”, it is recommended to acquire the knowledge about SARS-Cov-2 transmission and improve cognitive ability (57). 2) For the symptoms of “sad mood” and “amused”, it is recommended to develop new interests and get social support from family or friends, and keep close contact with them. Previous research confirms that family support can effectively intervene in mental health problems (58), and family separation has negative impacts on mental health (59). 3) To reduce the symptoms “relax” and “annoyed”, appropriate exercises at home are recommended, which may strengthen the immune system and effectively alleviate mood disorders (60).

The purpose of the current research is to explore possible potential pathways for intervention to address college students' mental health needs in pandemic isolation. To the best of our knowledge, this is the first network analysis on irritability, anxiety and depression among college students during the COVID-19 pandemic. In addition, we assessed the importance of each symptom in the comorbidity network by using strength centrality. This is also the first time that bridge centrality is computed in a network analysis of irritability, depression and anxiety, which is a more objective assessment of bridge nodes. The CS-coefficients of node strength and bridge strength are both 0.75. The estimations of node strength and bridge strength are sufficiently stable.

Despite these strengths, this study also has several limitations. First, the items of irritability, depression and anxiety used in this study do not share one-to-one match with those in previous network studies of anxiety and depression, leading to inconsistent results in this network with those in previous studies (48, 50). In addition, it is unclear whether symptom changes will affect the stability of the core symptoms. It should be noted that symptoms in PHQ-9 and GAD-7 do not cover all anxiety and depression symptoms; therefore, future research should consider a wider range of symptoms in order to identify the stability of central node in comorbidity networks of anxiety and depression. There are two possible reasons for the change in the network model. One reason is that the different measurement tools may contain different items. For example, there are differences between items of BDI-II and PHQ-9, or between those of BAI and GAD-7. Studies of comorbidity network for major depressive disorder show that depressive symptoms are not the most central symptoms in the network (61), inconsistent with the results of previous studies (50). Whether variation of items will change the comorbidity network requires further study. The second reason is that participant discrepancy exists, such as ethnic and cultural differences (62). Different participants have different sample characteristics. For example, patients with depression and eating disorders have different physical symptoms (63). In addition, as the study only includes a sample of college students isolated in the COVID-19 pandemic, the results may not be generalized to college students in non-pandemic isolation areas. The results of this study may only be applicable to a sample population that has been isolated for a period of time during the COVID-19 pandemic. In short, different measurement tools and participant discrepancy may affect the comparison of symptoms of the same mental disorders and the stability of the research results, especially the stability of the central node. As the network analysis is still in the early stages of development, the results should be cautiously interpreted. The cross-sectional design of this study makes it difficult to determine the continuity of the results; therefore, we plan to conduct more dynamic network analysis in the future research.

## 5. Conclusion

We used network analysis to explore network structure among irritability, depression and anxiety symptoms in Chinese college students during the COVID-19 pandemic. A strong interconnection and clusters were found within each disorder. We found that anxiety symptoms and irritability symptoms are core symptoms in the current network, such as “panic”, “annoyed” and “relax”. Interventions targeting these symptoms may be beneficial for the mental health of university students during COVID-19 pandemic. Future research should focus on the long-term effects of COVID-19 on mental health.

## Data Availability

The raw data supporting the conclusion of this article and will be made available by the authors, without undue reservation. Request to access this dataset should be directed to Li K, risyaiee@msn.cn.
