# Supplementary material for: The relationship between irritability, depression and anxiety among Chinese college students during the COVID-19 pandemic: A network analysis
Source: Front Child Adolesc Psychiatry. 2023 Apr 6;2:1045161. doi: 10.3389/frcha.2023.1045161 (PMC11732130; doi:10.3389/frcha.2023.1045161)
Supplement: Supplementary file 1 [file Datasheet1.docx]

**Supplementary Materials**

**The relationship between irritability, anxiety and depression among Chinese college students during the COVID-19 pandemic: A network analysis**

1. Table S1. Nonparametric Spearman rho correlation matrix of the variables selected for the network analysis
2. Fig. S1. Accuracy of edge weights
3. Fig. S2. Bootstrapped difference test for edge weights
4. Fig. S3. Stability of node strengths
5. Fig. S4. Bootstrapped difference test for node strengths
6. Fig. S5. Stability of node bridge strengths
7. Fig. S6. Bootstrapped difference test for node bridge strengths

Table S1. Nonparametric Spearman rho correlation matrix of the variables selected for the network analysis

|  | D1 | D2 | D3 | D4 | D5 | A1 | A2 | A3 | A4 | A5 | I1 | I2 | I3 | I4 | O1 | O2 | O3 | O4 |
| --- | --- | --- | --- | --- | --- | --- | --- | --- | --- | --- | --- | --- | --- | --- | --- | --- | --- | --- |
| D1 | 1.00 |  |  |  |  |  |  |  |  |  |  |  |  |  |  |  |  |  |
| D2 | 0.35 | 1.00 |  |  |  |  |  |  |  |  |  |  |  |  |  |  |  |  |
| D3 | 0.52 | 0.31 | 1.00 |  |  |  |  |  |  |  |  |  |  |  |  |  |  |  |
| D4 | 0.16 | 0.15 | 0.21 | 1.00 |  |  |  |  |  |  |  |  |  |  |  |  |  |  |
| D5 | 0.33 | 0.21 | 0.38 | 0.17 | 1.00 |  |  |  |  |  |  |  |  |  |  |  |  |  |
| A1 | 0.41 | 0.28 | 0.42 | 0.15 | 0.42 | 1.00 |  |  |  |  |  |  |  |  |  |  |  |  |
| A2 | 0.19 | 0.27 | 0.19 | 0.19 | 0.16 | 0.20 | 1.00 |  |  |  |  |  |  |  |  |  |  |  |
| A3 | 0.20 | 0.21 | 0.21 | 0.15 | 0.21 | 0.26 | 0.32 | 1.00 |  |  |  |  |  |  |  |  |  |  |
| A4 | 0.27 | 0.25 | 0.28 | 0.18 | 0.23 | 0.30 | 0.37 | 0.54 | 1.00 |  |  |  |  |  |  |  |  |  |
| A5 | 0.07 | 0.05 | 0.11 | 0.06 | 0.11 | 0.09 | ns | 0.07 | 0.10 | 1.00 |  |  |  |  |  |  |  |  |
| I1 | 0.27 | 0.20 | 0.26 | 0.17 | 0.26 | 0.26 | 0.30 | 0.25 | 0.41 | 0.06 | 1.00 |  |  |  |  |  |  |  |
| I2 | 0.23 | 0.22 | 0.27 | 0.15 | 0.22 | 0.25 | 0.29 | 0.30 | 0.42 | ns | 0.57 | 1.00 |  |  |  |  |  |  |
| I3 | 0.23 | 0.19 | 0.27 | 0.17 | 0.21 | 0.23 | 0.22 | 0.27 | 0.36 | 0.05 | 0.41 | 0.44 | 1.00 |  |  |  |  |  |
| I4 | 0.33 | 0.25 | 0.32 | 0.13 | 0.27 | 0.32 | 0.27 | 0.35 | 0.43 | ns | 0.45 | 0.48 | 0.47 | 1.00 |  |  |  |  |
| O1 | 0.20 | 0.16 | 0.21 | 0.09 | 0.20 | 0.25 | 0.21 | 0.22 | 0.31 | 0.07 | 0.36 | 0.42 | 0.39 | 0.43 | 1.00 |  |  |  |
| O2 | 0.24 | 0.16 | 0.25 | 0.11 | 0.22 | 0.27 | 0.22 | 0.22 | 0.35 | 0.10 | 0.41 | 0.44 | 0.40 | 0.45 | 0.53 | 1.00 |  |  |
| O3 | 0.24 | 0.13 | 0.28 | 0.09 | 0.24 | 0.24 | 0.11 | 0.11 | 0.15 | 0.13 | 0.17 | 0.17 | 0.20 | 0.17 | 0.19 | 0.18 | 1.00 |  |
| O4 | 0.19 | 0.18 | 0.19 | 0.09 | 0.15 | 0.22 | 0.23 | 0.22 | 0.34 | ns | 0.39 | 0.36 | 0.36 | 0.38 | 0.45 | 0.44 | 0.16 | 1.00 |

Note: The depression item: D1= sad mood; D2= appetite; D3= amused; D4= sleep; D5= anhedonia. The anxiety item: A1= relax; A2= disgust; A3= nervous; A4= panic; A5= ease. The inward irritability item: I1= self-hurt; I2= self-harm; I3= angry; I4= annoyed. The outward irritability item: O1= rough; O2= aggressive; O3= patient; O4= upset.

ns = nonsignificant, *p* < 0.05

Fig. S1. Accuracy of edge weights

*Note*: The red line depicts the sample edge weights and the gray bar depicts the bootstrapped confidence interval.

Fig. S2. Bootstrapped difference test for edge weights

*Note*: Gray boxes indicate edge weights that do not differ significantly from one another, while black boxes indicate edge weights that do differ significantly. Blue and red boxes on the diagonal correspond to edge weights with positive and negative correlations, respectively.

Fig. S3. Stability of node strengths

*Note*: The red bar represents the average correlation between strength in the full sample and subsample with the red area depicting the 2.5th quantile to the 97.5th quantile.

Fig. S4. Bootstrapped difference test for node strengths

*Note*: Gray boxes indicate node strengths that do not differ significantly from one another, while black boxes indicate node strengths that do differ significantly. The number in the white boxes (i.e., diagonal line) represent the value of node strengths.

Fig. S5. Stability of node bridge strengths

*Note*: The red bar represents the average correlation between bridge strength in the full sample and subsample with the red area depicting the 2.5th quantile to the 97.5th quantile.

Fig. S6. Bootstrapped difference test for node bridge strengths

*Note*: Gray boxes indicate node bridge strengths that do not differ significantly from one another, while black boxes indicate node bridge strengths that do differ significantly.
